# Epithelial and Mesenchymal Markers in Adrenocortical Tissues: How Mesenchymal Are Adrenocortical Tissues?

**DOI:** 10.3390/cancers13071736

**Published:** 2021-04-06

**Authors:** Iuliu Sbiera, Stefan Kircher, Barbara Altieri, Martin Fassnacht, Matthias Kroiss, Silviu Sbiera

**Affiliations:** 1 Department of Internal Medicine I, Division of Endocrinology and Diabetes, University Hospital Würzburg, 97080 Würzburg, Germany; e_sbiera_i@ukw.de (I.S.); Altieri_B@ukw.de (B.A.); Fassnacht_M@ukw.de (M.F.); 2 Institute for Pathology, University of Würzburg, 97080 Würzburg, Germany; stefan.kircher@uni-wuerzburg.de; 3 Clinical Chemistry and Laboratory Medicine, University Hospital Würzburg, 97080 Würzburg, Germany; 4 Comprehensive Cancer Center Mainfranken, University of Würzburg, 97080 Würzburg, Germany; 5 Department of Internal Medicine IV, University Hospital Munich, Ludwig-Maximilians-Universität München, 80336 Munich, Germany

**Keywords:** adrenocortical tissues, EMT, epithelial markers, mesenchymal markers, recurrence-free survival

## Abstract

**Simple Summary:**

Recent studies have hinted to an involvement of epithelial to mesenchymal transition, a mechanism often associated with metastasis in epithelial cancers, in adrenocortical carcinoma. We assessed, in a large number of normal, benign and malignant adrenocortical tissues, the expression of canonical epithelial and mesenchymal markers and compared it with their expression in typical epithelial and mesenchymal tissues. Surprisingly, both normal and neoplastic adrenocortical tissues lacked expression of epithelial markers but strongly expressed mesenchymal markers, suggesting a higher similarity of adrenocortical tissues to mesenchymal compared to epithelial tissues, reminiscent of the adrenocortical origin from the intermediate mesoderm. Despite their ubiquitous expression in all adrenocortical tissues, mesenchymal markers had a variable expression in ACC, associating either directly or inversely with different clinical markers of tumor aggressiveness. Our data are an important step in better understanding the adrenocortical tissues in general and adrenocortical tumorigenesis in particular, and could be exploited therapeutically in the future.

**Abstract:**

A clinically relevant proportion of adrenocortical carcinoma (ACC) cases shows a tendency to metastatic spread. The objective was to determine whether the epithelial to mesenchymal transition (EMT), a mechanism associated with metastasizing in several epithelial cancers, might play a crucial role in ACC. 138 ACC, 29 adrenocortical adenomas (ACA), three normal adrenal glands (NAG), and control tissue samples were assessed for the expression of epithelial (E-cadherin and EpCAM) and mesenchymal (N-cadherin, SLUG and SNAIL) markers by immunohistochemistry. Using real-time RT-PCR we quantified the alternative isoform splicing of FGFR 2 and 3, another known indicator of EMT. We also assessed the impact of these markers on clinical outcome. Results show that both normal and neoplastic adrenocortical tissues lacked expression of epithelial markers but strongly expressed mesenchymal markers N-cadherin and SLUG. FGFR isoform splicing confirmed higher similarity of adrenocortical tissues to mesenchymal compared to epithelial tissues. In ACC, higher SLUG expression was associated with clinical markers indicating aggressiveness, while N-cadherin expression inversely associated with these markers. In conclusion, we could not find any indication of EMT as all adrenocortical tissues lacked expression of epithelial markers and exhibited closer similarity to mesenchymal tissues. However, while N-cadherin might play a positive role in tissue structure upkeep, SLUG seems to be associated with a more aggressive phenotype.

## 1. Introduction

Adrenocortical carcinoma (ACC) is a rare endocrine malignancy and its pathogenesis is poorly understood. Complete surgical resection is the treatment of choice in localized ACC and is virtually the only option to achieve a cure. As recurrence is frequent, adjuvant therapy is recommended in most patients [1,2,3,4]. Several genomic studies have been performed in adrenocortical tumors with the goal to better understand the mechanisms that lead to tumorigenesis, hormone excess and malignancy [5,6,7,8]. Using clustering of genome wide data, these studies consistently identified a subgroup of highly malignant tumors characterized by enhanced genomic variability and altered gene expression [9,10]. 

In irresectable and metastatic disease, cytotoxic chemotherapy is the standard treatment. The first and largest randomized phase III study in advanced ACC established etoposide, doxorubicin, cisplatin plus mitotane (EDP-M) as the cytotoxic chemotherapy of first choice in metastatic ACC [11]. With a median progression-free survival of only 5.0 months and an overall survival of only 14.8 months in the group receiving EDP-M, the prognosis is still poor. In the meantime, several other therapeutic approaches have been investigated [12,13,14,15,16], but a clinically meaningful breakthrough has not yet been achieved. 

**Figure 1:**
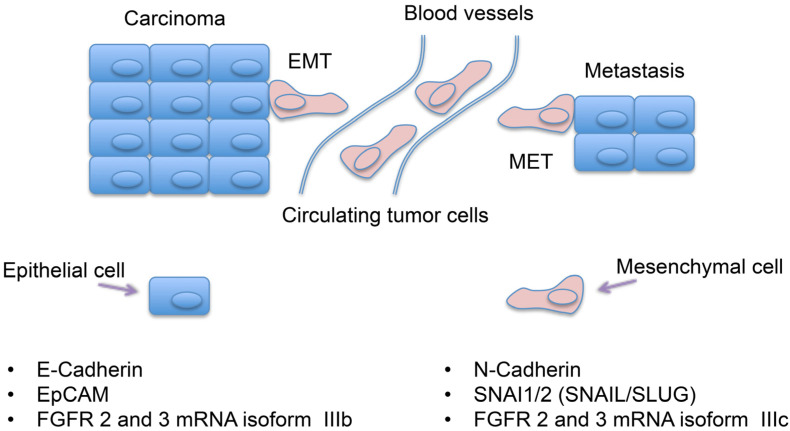
Classical EMT in cancer cells. Upper panel: EMT (Epithelial to Mesenchymal Transition) and MET (Mesenchymal to Epithelial Transition) processes in metastatic spread. Lower panel: Canonical markers of epithelial (left) and mesenchymal (right) cells.

Epithelial-mesenchymal transition (EMT) is a process that was first recognized as a feature of embryogenesis and, together with its reverse process MET (mesenchymal-epithelial transition), plays a crucial role in the development of many tissues and organs [17,18]. Most importantly, the mechanism of EMT, which allows the epithelial tumor cells to acquire a motile mesenchymal phenotype [19], is diverted by several types of cancer to promote metastasis and resistance to treatment [20]. This process has been considered to be implicated in metastatic spread of such a large variety of human cancers like breast, prostate, lung etc. [21,22,23] (Figure 1). 

Around 90% of all malignancies originate from epithelial tissue. The adrenocortical tissue is also classically categorized as an epithelial tissue. Studies on the adrenal cortex place its origins in the intermediate mesoderm (mesenchymal) [24], but it is considered to have undergone MET to an epithelial tissue [25]. Accordingly, adrenal tumors are also classified as carcinomas (tumors of an epithelial tissue) [26] as opposed to sarcomas (tumors of a mesenchymal tissue) [27]. Two studies have provided a first indication that adrenocortical tissues are expressing some mesenchymal markers [28,29]. However, the number of adrenocortical carcinoma tissues analyzed in these studies was low (24 cases in each study) and a correlation between EMT marker expression and clinicopathological markers indicative of tumor aggressiveness was not possible.

A number of distinct molecular processes participate in EMT like activation of transcription factors, expression of specific cell-surface proteins, reorganization and expression of cytoskeletal proteins etc. In many cases, the factors involved are also used as biomarkers to demonstrate that a specific cell is undergoing EMT [20]. For example, E-cadherin and epithelial cell adhesion molecule (EpCAM) are considered as classical epithelial markers while N-cadherin, SNAIL and SLUG are considered mesenchymal markers [30]. In addition, at mRNA level, the expression of the epithelial (IIIB) and mesenchymal (IIIC) isoforms of FGFR 2 and 3 can be also used to characterize EMT [31] (Figure 1).

Epithelial cell adhesion molecule (EpCAM) is a transmembrane glycoprotein mediating Ca2+-independent homotypic cell–cell adhesion in epithelia [32] associating with the actin cytoskeleton via an intermediate molecule [33]. Epithelial tumors are often characterized by strong expression of EpCAM while its expression is downregulated during EMT but then upregulated once the metastasis reaches its future tumor site, where the MET process is supposed to take place [34].

E-cadherin and N-cadherin are classical cadherins and share similar structures. They form cadherin-catenin complex where the cytoplasmic domain consists of EC repeats that bind with catenins to moderate the cytoskeletal filament containing actin. The structural difference between E-cadherin and N-cadherin is that E-cadherin binds with the shorter isoform of p120 catenin while N-cadherin binds with the longer isoform and the switch from E-cadherin expression to N-cadherin, which mediates weaker cell-cell interactions is classically used as a mesenchymal marker to define EMT [35]. N-cadherin is also present in few epithelial tissues such as hepatocytes but only together with a much stronger E-cadherin expression [30]. 

Snail and Slug (SNAI1 and SNAI 2), are two transcription factors that suppress E-cadherin and lead to a decrease in cell-to-cell adhesion and are also commonly used to detect EMT [36,37]. Knockout models for both SNAIL and SLUG showed significant reduction in cancer invasiveness [38,39].

At mRNA level, the fibroblast growth factors receptors (FGFR) isoform switching is another model that can be used as a marker for EMT. Fibroblast growth factor receptors (FGFRs) are a family of receptor tyrosine kinases expressed on the cell membrane that play crucial roles in both developmental and adult cells. The fibroblast growth factor receptor family has 4 members, FGFR1, FGFR2, FGFR3, and FGFR4 [40]. The FGFRs consist of three extracellular immunoglobulin-type domains (D1-D3), a single-span trans-membrane domain and an intracellular split tyrosine kinase domain. FGFs interact with the D2 and D3 domains, with the D3 interactions primarily responsible for ligand-binding specificity [40]. The receptors 1 to 3 have the unique feature of having two isoforms due to alternative splicing of the D3 domain which changes the specificity [41]. For the FGFRs 2 and 3 it has been shown that the isoform IIIB is mainly present in epithelial cells while the isoform IIIC is mostly mesenchymal [31,42]. 

We hypothesized that EMT may be relevant for the subgroup of highly aggressive ACCs and investigated the expression of several EMT markers in a large cohort of adrenocortical carcinoma tissue samples (including also normal adrenals and benign adrenocortical tumors) and correlated them with clinical features and patient outcome.

## 2. Materials and Methods

### 2.1. Patient Material

Formalin fixed, paraffin embedded (FFPE) tissue samples of ACC (*n* = 138; a total of 122 samples were previously assembled in seven tissue microarrays/TMAs [43]), adrenocortical adenomas (ACA, *n* = 29), as well as normal adrenal glands resulting from kidney cancer surgery (NAG, *n* = 3) were analyzed. All patients gave informed consent and the study was approved by the ethical committee of the University of Würzburg (88/11). This cohort was clinically annotated and the data were collected through the registry of the European Network for the Study of Adrenal Cancer (ENSAT). A short clinical description of the patients can be found in Table 1. For establishment of the detection of the different epithelial markers we used tissues from two normal thyroid, three thyroid carcinoma and three colon carcinoma. For establishment of the detection of the different mesenchymal markers we used tissues from one osteosarcoma, one liposarcoma, one leiomyosarcoma and one pleomorphic sarcoma.

### 2.2. Immunohistochemistry

The immunohistochemistry procedure is explained in more detail elsewhere [43]. In short, the FFPE TMA and full tissue slices of ~2 µm thickness were mounted on SuperFrost glass slides (Langenbrinck, Emmendingen, Germany), deparaffinized in xylene, and rehydrated in a series of water in alcohol dilutions. Antigen retrieval was achieved by boiling in 1 mM citrate buffer (pH 6.5) inside a pressure cooker for 13 min. Endogenous peroxidase was blocked with a 3% solution of hydrogen peroxide in methanol for 10 min, followed by the blocking of non-specific binding for another 10 min with the help of a 20% solution of human AB serum in PBS. The primary antibodies used were: E-Cadherin (Sigma-Aldrich, St. Louis, MO, USA, mouse monoclonal, clone CL1172, 1:2250 dilution), EpCAM (abcam, Cambridge, UK, rabbit polyclonal (ab71916), 1:20000 dilution), N-Cadherin (Santa Cruz Biotechnology, Dallas, TX, USA, mouse monoclonal, clone D-4, 1:125 dilution), SLUG (Novus Biologicals, Centennial, CO, USA, mouse monoclonal, clone OTI1A6, 1:300 dilution) and SNAIL (kindly provided as a gift from Dr. A. García de Herreros, University Pompeu Fabra, Barcelona, Spain; clone EC3, subclone EC11, 1:50 dilution [47]). Incubation time for the primary antibodies was 1 h at room temperature in PBS. Signal amplification was done with “HiDef Detection HRP Polymer System” (Cell Marque, Rocklin, CA, USA) and the signal was developed using the chromogen DAB substrate kit (Cell Marque) for 10 min. Counterstaining of nuclei was performed using Meyer’s Hematoxylin for 2 min (Carl Roth, Karlsruhe, Germany), followed by washing in running tap water for 5 min. After dehydration, slides were mounted using Entellan (Merck, Darmstadt, Germany) and borosilicate glass coverslips (A. Hartenstein, Würzburg, Germany).

Stained tissue slides were imaged with the Leica Aperio Versa brightfield scanning microscope (Leica, Wetzlar, Germany) and evaluated using a semi-quantitative H-Score [48] that estimated the intensity of the staining [scored as negative (0), low (1), medium (2) and high (3)] and the percentage of positive cells (scored as 0, 0.1, 0.5, or 1 if 0%, 1–9%, 10–49%, or ≥50% of the cells were positive, respectively). All slides were evaluated by two independent investigators blinded to clinical data. Low expression was defined as H-score < 2 and high score as H-score ≥ 2.

### 2.3. Cell Culture

NCI-H295R cells were obtained from ATCC and cultured in DMEM/F12 supplemented with 1× Insulin-Transferrin-Selenium and Nu-Serum (2.5%). CU-ACC1 and CU-ACC2 cells were obtained from Katja Kiseljak-Vassiliades and cultured as described [46]. In brief, a 1:3 mixture of F12 Ham and DMEM high glucose (both Thermo Fisher Scientific, Waltham, MA, USA) was supplemented with 10% FCS, 0.4 μg/mL hydrocortisone (Sigma-Aldrich), 5 μg/mL insulin (Sigma-Aldrich), 8.4 ng/mL cholera toxin (Sigma-Aldrich), 24 μg/mL adenine (Sigma-Aldrich) and 10 ng/mL EGF (Thermo Fisher Scientific). MUC-1 were obtained from Constanze Hantel and cultured in DMEM Advance 1% penicilin/streptomycin and 10% FCS as described [45].

### 2.4. Real-Time PCR

RNA was first extracted from frozen tissues using the RNeasy Lipid Tissue Kit (Qiagen, Düsseldorf, Germany) and reverse transcribed using the High-Capacity cDNA Reverse Transcription Kit (Thermo Fisher Scientific). TaqMan Gene Expression Assay (Thermo Fisher Scientific) was used with the Hs01005396_m1 (FGFR3 IIIB) and Hs00997397_m1 (FGFR3 IIIC) probes and the following custom primers/probes for FGFR2 receptor isoforms [49]:

FGFR2 IIIb fw: 5’-GGCTCTGTTCAATGTGACCGA-3′; rev: 5′-GTTGGCCTGCCCTATATAATTGGA-3′; TaqMan probe: 5′-TTTCCCCAGCATCCGCC-3′ 

FGFR2 IIIc up: 5′-CACGGACAAAGAGATTGAGGTTCT-3′; low: 5′-CCGCCAAGCACGTATATTCC-3′; TaqMan probe: 5′-CCAGCGTCCTCAAAAG-3′

The expression of β-actin (Hs9999903_m1) was used for normalization. Amplification and results evaluation were performed using a Bio-Rad CFX-96 Dx system (Bio-Rad Laboratories, Hercules, CA, USA).

### 2.5. Statistical Analyses

The relationship between two categorical variables was determined using the Chi square test. For non-parametrical multiple comparisons between groups the Kruskal-Wallis test with Dunn’s post-hoc comparison was used. A *p*-value < 0.05 was considered statistically significant. *p*-values between 0.05 and 0.15 were considered indicative of a statistical trend. All statistical analyses were performed with Graph Pad Prism v 7 for Windows (GrapPad Software Inc., La Jolla, CA, USA). For ACC patients, the Kaplan-Meier method was used to estimate overall survival (OS, in all patients with primary tumors) and recurrence-free survival (RFS, in patients with complete resection of the primary tumor) using IBM SPSS v 26 for Windows (SPSS Inc., Chicago, IL, USA). After ≈10% of subjects remaining at risk the Kaplan-Meier survival was curtailed.

## 3. Results

### 3.1. Typical Epithelial Adhesion Markers Are Not Expressed in Adrenocortical Tissues

**Figure 2:**
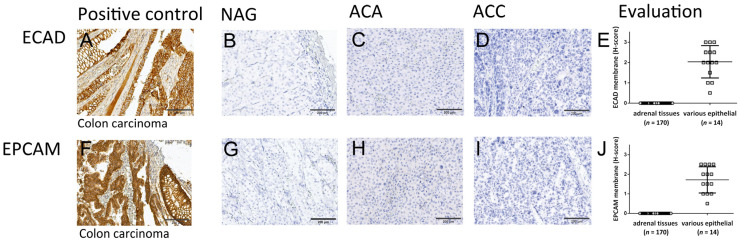
Expression of canonical immunohistochemical epithelial markers in adrenocortical tissues. Staining of epithelial markers E-cadherin (**A**–**E**) and Epithelial Cell Adhesion Molecule (EpCAM) (**F**–**J**) protein in classical epithelial tissues (**A**,**F**) vs. normal adrenal glands (NAG, *n* = 3; **B**,**G**) vs. adrenocortical adenomas (ACA, *n* = 29; **C**,**H**) vs. adrenocortical carcinomas (ACC, *n* = 138; **D**,**I**). Scale bar = 200 µm. Quantitative evaluation in (**E**,**J**), respectively.

The expression of E-cadherin, was absent in all adrenal tissues analyzed (*n* = 170), both normal and tumoral (Figure 2B–E), while this marker showed a normal, membrane expression in 14 different epithelial tissues analyzed (Figure 2A,E). Similar results were also observed for EpCAM, which was also completely missing in all adrenocortical tissues analyzed (Figure 2G–J) while strong expression of this marker in classical epithelial tissues was observed that were used as positive controls (Figure 2F,J).

### 3.2. Adrenocortical Tissues Are Characterized by Relatively High Expression of Mesenchymal Markers

**Figure 3:**
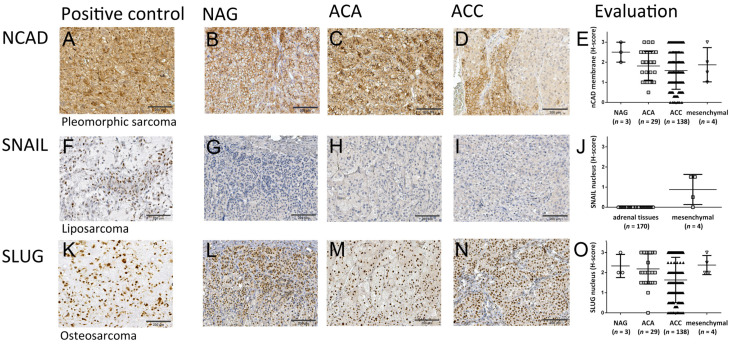
Expression of canonical immunohistochemical mesenchymal markers in adrenocortical tissues. Staining of mesenchymal markers N-cadherin (**A**–**E**), Zinc finger protein SNAI1 (SNAIL) (**F**–**J**) and Zinc finger protein SNAI2 (SLUG) (**K**–**O**) in classical mesenchymal cancers (**A**,**F**,**K**) vs. normal adrenal glands (NAG, *n* = 3; **B**,**G**,**L**) vs. adrenocortical adenomas (ACA, *n* = 29; **C**,**H**,**M**) and vs. adrenocortical carcinomas (ACC, *n* = 138; **D**,**I**,**N**). Scale bar = 200 µm. Quantitative evaluation in (**E**,**J**,**O**), respectively.

Surprisingly, membrane N-cadherin expression was expressed in normal adrenocortical tissues at high levels (H-score 2.5 ± 0.5, Figure 3B,E). The expression was distributed rather equally between the three functional regions, with slightly lower expression in the zona fasciculata (Supplementary Figure S1A–C). Most adrenocortical adenomas and carcinomas demonstrated moderate to high expression (ACA mean H-score 1.8 ± 0.7; Figure 3C,E, ACC 1.6 ± 0.9; Figure 3D,E) similar to the mesenchymal sarcomas (1.9 ± 0.8; Figure 3A,E). There were no significant differences between the different adrenocortical tissues, only a trend (NAG vs. ACA: *p* = 0.14, NAG vs. ACC: *p* = 0.09 and ACA vs. ACC: *p* = 0.20), however, interestingly, the variability of expression of N-cadherin increased gradually from NAG to ACA and then to ACC (Figure 3E) as shown by increasing coefficients of variation (NAG 20.00%, ACA 39.25% and ACC 58.53%; mesenchymal 45.54%).

While Snail nuclear expression was found in most mesenchymal tissues tested (Figure 3F,J), detectable expression was not observed in any of the adrenocortical tissues (Figure 3G–I and evaluation in Figure 3J). In contrast, a strong expression of Slug was found in both mesenchymal tissues (mean H-score 2.3 ± 0.5; Figure 3K,O) and normal and benign adrenal tissues without statistically significant differences among groups (NAG mean H-score 2.3 ± 0.5, ACA 2.2 ± 0.7; Figure 3L,M,O) but variable expression in ACC (mean H-score 1.6 ± 1.1; Figure 3N,O). 

Only the expression in ACC was significantly different compared to the other two adrenocortical sample sets (NAG vs. ACA: *p* = 0.79, NAG vs. ACC: *p* = 0.02 and ACA vs. ACC: *p* = 0.01*) but as with N-cadherin, the variability of expression of SLUG increased gradually from NAG to ACA and then to ACC (Figure 3O) as shown by increasing coefficients of variation (NAG 24.74%, ACA 34.29% and ACC 68.77%; mesenchymal 20.16%). Interestingly, in the normal adrenal gland tissue the most nuclei stained positive were localized in the subcapsular region, in the zona glomerulosa (Supplementary Figure S1D–F).

### 3.3. FGFR1-3 Isotype Expression Shows a Pattern Similar to Mesenchymal Tissues

**Figure 4:**
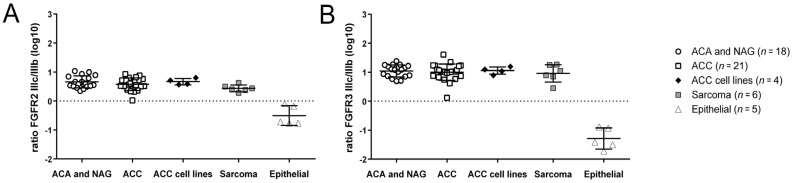
Differential expression of FGFR splice variants mRNA in adrenocortical tissues. Analysis of the ratios between the “mesenchymal” (IIIC) and “epithelial” (IIIB) splice variants for FGFR-2 (**A**) and 3 (**B**), in normal adrenal glands (NAG), adrenocortical adenomas (ACA) and carcinomas (ACC) as compared to mesenchymal sarcomas and canonical epithelial tissues; for better visualization of the isoform switch, results are represented in log10 base.

To further elucidate the epithelial vs. mesenchymal phenotype of adrenocortical tumors, we used the ratio between the “mesenchymal” IIIC and the “epithelial” IIIB isotypes of FGFR 2-3 in a subgroup of fresh frozen adrenocortical tissue samples and cell lines. Isoform IIIC of FGFR 2 was expressed on average 4.6 times higher than IIIB in all adrenocortical tissues studied (Figure 4A) (ratio IIIC/IIIB: 5.1 ± 2.6 for the normal adrenal glands and adrenocortical adenomas vs. 4.2 ± 2 for the ACC samples vs. 4.8 ± 1.2 for ACC cell-lines) similar to the mesenchymal sarcomas (2.8 ± 0.8), but in contrast to the epithelial samples where the IIIB isoform was higher expressed than the IIIC isoform, as expected (ratio IIIB/IIIC: 3.9 ± 2.3). For FGFR 3 the IIIC/IIIB ratios were even higher (Figure 4B) (12.2 ± 5.5 for the normal adrenal glands and adrenocortical adenomas vs. 11.9 ± 7.7 for the ACC samples vs. 11.7 ± 3.2 for ACC cell-lines) similar again to the mesenchymal sarcomas (9.1 ± 7.1). The epithelial control tissues showed again, as expected, higher IIIB than IIIC expression (ratio IIIB/IIIC: 25.1 ± 18.4).

### 3.4. SLUG and N-Cadherine Are Associated in an Opposite Manner with Pathoclinical Tumor Aggressiveness Parameters 

**Figure 5:**
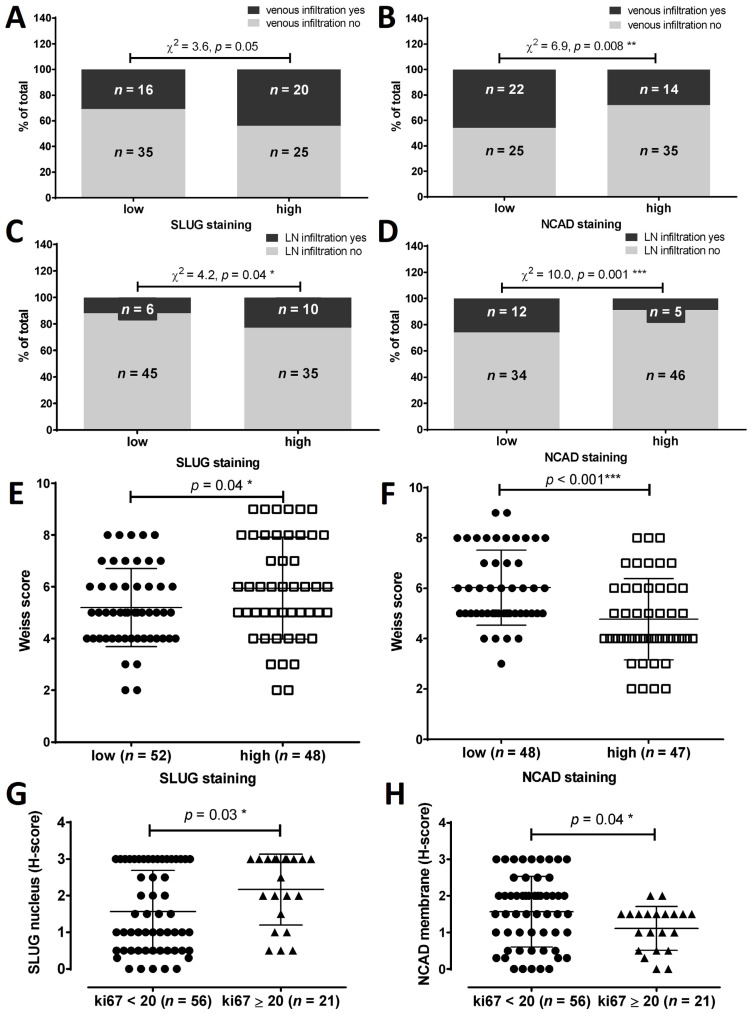
Comparison between relevant clinicopathological data and expression levels of mesenchymal markers SLUG and N-Cadherin. (**A**,**B**) venous tumor infiltration, (**C**,**D**) lymph node tumor infiltration, (**E**,**F**) Weiss score distribution and (**G**,**H**) proliferation marker Ki67. "*n*" numbers represent the absolute number of cases in each subgroup. χ^2^ analyses have been performed between proportions (%) in each staining intensity group. * *p <* 0.05, ** *p <* 0.01, *** *p <* 0.001.

Since expression of NCAD and SLUG showed an increase in variability from normal, to benign, to malignant adrenocortical tissues, this suggested a modulation of these factors during the tumorigenesis and tumor progression. Therefore we looked for possible associations between different expression levels of NCAD and SLUG and indicators of tumoral metastatic potential. The presence of venous infiltration was associated with high (H-score ≥ 2) vs. low (H-score < 2) expression of SLUG (31 vs. 44%, χ^2^ = 3.6, *p* = 0.05) (Figure 5A), but with lower expression of N-cadherin (28 vs. 46%, χ^2^ = 6.9, *p* = 0.008) (Figure 5B). Similarly, lymph node infiltration was significantly more often present in tumors with high SLUG expression (23% vs. 12%, χ^2^ = 4.2, *p* = 0.04) (Figure 5C) and with low N-cadherin expression (26% vs. 9%, χ^2^ = 10.0, *p* = 0.001) (Figure 5D). Unsurprisingly, also the mixed pathomorphological diagnostic Weiss score, an indicator for tumor malignancy, was significantly higher for samples with low NCAD expression (6.0 ± 1.5 vs. 4.7 ± 1.6, *p* < 0.001) (Figure 5F), and for samples with high SLUG expression (6.0 ± 1.9 vs. 5.1 ± 1.5, *p* = 0.04) (Figure 5E). A Mann-Whitney test of the distribution of N-cadherin and SLUG expression in tumors with low and high expression of the proliferation marker Ki67, the best defined prognostic marker for the ACC [50], confirmed this association. In tumors with high Ki67 expression the SLUG expression was significantly higher (2.2 ± 0.9 vs. 1.5 ± 1.1, *p* = 0.03) (Figure 5G) while the expression of N-cadherin was significantly lower (1.1 ± 0.6 vs. 1.5 ± 0.9, *p* = 0.04) (Figure 5H). 

### 3.5. SLUG and N-Cadherin Expression Have a Divergent Association with ACC Patients’ Progression-Free Survival 

**Figure 6:**
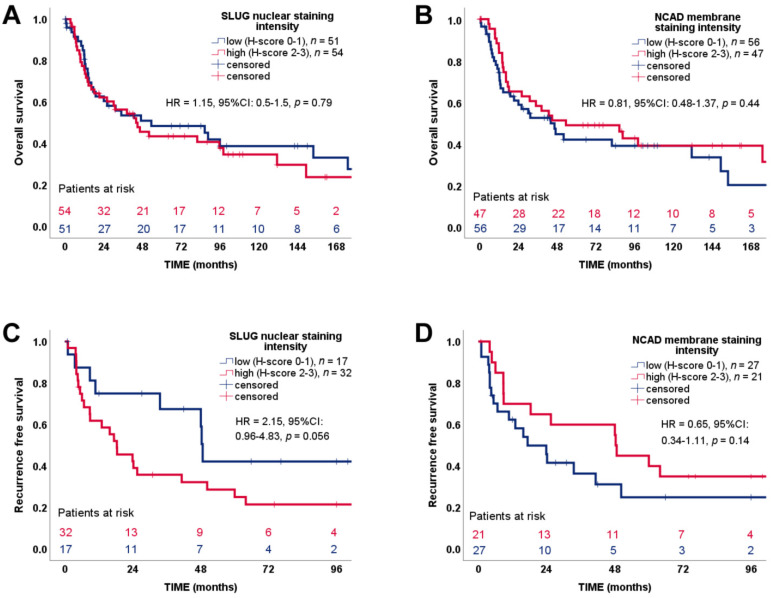
Correlation of patient survival with expression of mesenchymal markers SLUG and N-Cadherin. (**A**,**B**) overall survival (**C**,**D**) recurrence-free survival.

We next investigated a potential association of SLUG and N-cadherin expression with patient outcome and found no difference on OS (low vs. high SLUG expression: Average survival time 64.20 ± 10.27 vs. 68.82 ± 9.14 months, HR = 1.15, 95%CI: 0.5–1.5, *p* = 0.79 and low vs. high N-cadherin expression: Mean survival time 65.11 ± 9.49 vs. 71.22 ± 10.66 months, HR = 0.81, 95%CI: 0.48–1.37, *p* = 0.44) (Figure 6A,B) and only a trend that high SLUG expression correlated with a less favorable RFS in ACC patients after complete resection (high vs. low SLUG expression: Mean survival time 25.96 ± 5.40 vs. 49.82 ± 10.12 months, HR = 2.15, 95% CI: 0.96–4.83, *p* = 0.056) (Figure 6C). For N-cadherin the situation was opposite, while again not statistically significant, there was a light trend that high N-cadherin expression correlated with a better progression-free survival (mean survival time 40.12 ± 8.35 vs. 21.32 ± 6.6 months, HR = 0.65, 95% CI: 0.34–1.11, *p* = 0.14) (Figure 6D).

## 4. Discussion

In this study we investigated a series of both classical epithelial and mesenchymal markers in a large cohort of normal, benign and malignant adrenocortical tissues, and compared the expression of these markers with that in epithelial and mesenchymal control tissues. Against our hypothesis, our analysis revealed that in adrenocortical tumors EMT indicated by a more frequent occurrence of mesenchymal markers in neoplastic tissue, does not appear to play a role in tumor progression as suggested before in smaller studies [28,29]. Adrenocortical tissues do not express established epithelial markers like E-cadherin and EpCAM but express a series of “classical” mesenchymal markers like Slug and N-cadherin at similar levels as mesenchymal tissues. 

By using the more recently discovered marker of alternative mRNA splicing of the FGFR2 and 3 [42,51,52,53] we confirmed that adrenocortical tissues are more similar to mesenchymal than to epithelial tissues. This may be due to the special case of adrenocortical tissue as it originates during embryogenesis from the intermediate mesoderm, but is considered to undergo MET to result in an epithelial tissue [25]. Obviously, this epithelial transformation is incomplete and the adrenal cortex keeps most of its mesenchymal characteristics at molecular level.

While expressed in all adrenocortical tissues, there may still be a role of mesenchymal differentiation status in tumor aggressiveness. While higher SLUG expression is associated with more aggressive behavior of the tumors as indicated by its association with markers for lymphatic and hematogenic metastasizing and high cell proliferation, N-cadherin appears to play the role of major cell- cell adhesion molecule in the adrenocortical tissue and thus is a counterplayer of SLUG. There are also other tissues where N-cadherin is the prevalent constituent of adherens junctions such as neural tissues [54]. It is hence likely that adherens junctions in adrenocortical tissue are predominantly mediated by N-cadherin instead of the E-cadherin, which is more commonly found in epithelial adherens junctions. However, only future studies at a deeper molecular level on the cell-to-cell interactions in the adrenal will be able to definitely answer this question. 

Interestingly, SLUG nuclear expression in the normal adrenal gland was highest in the subcapsular area of the zona glomerulosa. This zone accommodates a subset of cells that have been reported to centripetally migrate towards the center of the gland and are responsible for the permanent renewal of the adrenocortical tissue [55]. The idea of a progenitor cell population that gives rise to all differentiated cell types within the adrenal cortex is old [56,57], but while its subcapsular localization has been clarified using animal models [58,59], there is not yet an universally accepted immunohistochemical marker that can be used to identify this population. It has been shown that Wnt, β-catenin and Shh all play an important role in this process [60,61,62], however, their expression in the adult adrenal cortex did not coincide with cell-proliferation markers [63] so they cannot be used to identify the progenitor cell population. The best candidate to date is the Notch atypical ligand Delta-like homologue 1 (DLK1) [64]. 

It would have been especially interesting to correlate in more detail the expression of SLUG and N-cadehrin in metastases with clinicopathological characteristics of the same, especially KI67. However, these data have been inconsistently retrieved in the past and the rarity of ACC renders the collection of such a larger series of clinically well-annotated cases prospectively quite challenging. Another limitation is the perceived limited choice of both epithelial and mesenchymal markers analyzed. While the list of possible specific markers is very long [20], we have concentrated on the best defined and used markers in each category. But we did not limit ourselves to immunohistological staining investigating also markers defined at mRNA level, thus covering quite a broad selection of pathways involved in cell adhesion, migration and response to external stimuli. The results of all these analyses corroborated with each other to give a synchronized picture on the role played by these markers in the adrenal tissues.

## 5. Conclusions

We could show that adrenocortical tissues, whether normal, benign or malignant, are characterized by lack of expression of classical epithelial tissues and are closer to mesenchymal tissues through high expression of classical mesenchymal markers like N-cadherin and SLUG. These factors also appear to play a role in cancer progression in ACC: While N-cadherin seems to have a positive role in the tissue structure sustainability and against metastatic spread, SLUG seems to promote this.

## Figures and Tables

**Table 1:** Patient clinical characteristics.

	Normal Adrenal Gland	ACA	ACC
*n*	3	29	138
Sex [male/female]	1/2	11/18	45/93
Age [yr (sd)]	49 (11)	51 (14)	50 (15)
Size of the tumor [cm (sd)]		3.3 (1.2)	10 (4.4)
Hormone secretion			
Cortisol—*n* (%)		11 (38%)	50 (37%)
Androgen—*n* (%)		0 (0%)	10 (7%)
Aldosterone—*n* (%)		7 (24%)	4 (3%)
Inactive—*n* (%)		11 (38%)	21 (15%)
Unknown—*n* (%)		0 (0%)	53 (38%)
Tumor localization—*n* (%)			
Primary—ENSAT stage I+II			44 (32%)
Primary—ENSAT stage III			37 (27%)
Primary—ENSAT stage IV			25 (18%)
Local recurrences			21 (15%)
Distant metastases			11 (8%)
Ki67 index [median (range)]			10 (1–70)
Weiss Score [median (range)]			5 (2–9)

For the evaluation of the FGFR 1-3 isoforms at mRNA levels, we used available frozen tissue from 18 ACA and NAG, 21 ACC, 6 sarcoma (2× osteosarcoma, 2× liposarcoma, 1× synovialsarcoma, 1× rhabdomyosarcoma), 5 epithelial tumors (3 colon carcinoma, 1 thyroid carcinoma, 1 ovarian carcinoma), as well as four different ACC cell lines (NCI-H295R [44], MUC-1 [45], CU-ACC1 and CU-ACC2 [46]).

## Data Availability

All data presented in this study is contained within the article and supplementary material.

## Supplementary Materials

The following are available online at https://www.mdpi.com/article/10.3390/cancers13071736/s1, Figure S1: Expression of canonical immunohistochemical mesenchymal markers in normal adrenal gland.

## References

[1] Fassnacht M., Dekkers O.M., Else T., Baudin E., Berruti A., de Krijger R., Haak H.R., Mihai R., Assie G., Terzolo M. (2018). European Society of Endocrinology Clinical Practice Guidelines on the management of adrenocortical carcinoma in adults, in collaboration with the European Network for the Study of Adrenal Tumors. Eur. J. Endocrinol..

[2] Fassnacht M., Assie G., Baudin E., Eisenhofer G., de la Fouchardiere C., Haak H.R., de Krijger R., Porpiglia F., Terzolo M., Berruti A. (2020). Adrenocortical carcinomas and malignant phaeochromocytomas: ESMO-EURACAN Clinical Practice Guidelines for diagnosis, treatment and follow-up. Ann. Oncol..

[3] Jasim S., Habra M.A. (2019). Management of Adrenocortical Carcinoma. Curr. Oncol. Rep..

[4] Else T., Kim A.C., Sabolch A., Raymond V.M., Kandathil A., Caoili E.M., Jolly S., Miller B.S., Giordano T.J., Hammer G.D. (2014). Adrenocortical carcinoma. Endocr. Rev..

[5] Assie G., Letouze E., Fassnacht M., Jouinot A., Luscap W., Barreau O., Omeiri H., Rodriguez S., Perlemoine K., Rene-Corail F. (2014). Integrated genomic characterization of adrenocortical carcinoma. Nat. Genet..

[6] Giordano T.J., Kuick R., Else T., Gauger P.G., Vinco M., Bauersfeld J., Sanders D., Thomas D.G., Doherty G., Hammer G. (2009). Molecular classification and prognostication of adrenocortical tumors by transcriptome profiling. Clin. Cancer Res..

[7] Zheng S.Y., Cherniack A.D., Dewal N., Moffitt R.A., Danilova L., Murray B.A., Lerario A.M., Else T., Knijnenburg T.A., Ciriello G. (2016). Comprehensive Pan-Genomic Characterization of Adrenocortical Carcinoma (vol 29, pg 723, 2016). Cancer Cell.

[8] Mohan D.R., Lerario A.M., Hammer G.D. (2018). Therapeutic Targets for Adrenocortical Carcinoma in the Genomics Era. J. Endocr. Soc..

[9] Jouinot A., Bertherat J. (2018). Management of endocrine disease: Adrenocortical carcinoma: Differentiating the good from the poor prognosis tumors. Eur. J. Endocrinol..

[10] Crona J., Beuschlein F. (2019). Adrenocortical carcinoma—Towards genomics guided clinical care. Nat. Rev. Endocrinol..

[11] Fassnacht M., Terzolo M., Allolio B., Baudin E., Haak H., Berruti A., Welin S., Schade-Brittinger C., Lacroix A., Jarzab B. (2012). Combination Chemotherapy in Advanced Adrenocortical Carcinoma. N. Engl. J. Med..

[12] Altieri B., Ronchi C.L., Kroiss M., Fassnacht M. (2020). Next-generation therapies for adrenocortical carcinoma. Best Pract. Res. Clin. Endocrinol. Metab..

[13] Cosentini D., Badalamenti G., Grisanti S., Basile V., Rapa I., Cerri S., Spallanzani A., Perotti P., Musso E., Lagana M. (2019). Activity and safety of temozolomide in advanced adrenocortical carcinoma patients. Eur. J. Endocrinol..

[14] Megerle F., Kroiss M., Hahner S., Fassnacht M. (2019). Advanced Adrenocortical Carcinoma-What to do when First-Line Therapy Fails?. Exp. Clin. Endocrinol. Diabetes.

[15] Henning J.E.K., Deutschbein T., Altieri B., Steinhauer S., Kircher S., Sbiera S., Wild V., Schlotelburg W., Kroiss M., Perotti P. (2017). Gemcitabine-Based Chemotherapy in Adrenocortical Carcinoma: A Multicenter Study of Efficacy and Predictive Factors. J. Clin. Endocrinol. Metab..

[16] Fassnacht M., Berruti A., Baudin E., Demeure M.J., Gilbert J., Haak H., Kroiss M., Quinn D.I., Hesseltine E., Ronchi C.L. (2015). Linsitinib (OSI-906) versus placebo for patients with locally advanced or metastatic adrenocortical carcinoma: A double-blind, randomised, phase 3 study. Lancet. Oncol..

[17] Thiery J.P., Acloque H., Huang R.Y., Nieto M.A. (2009). Epithelial-mesenchymal transitions in development and disease. Cell.

[18] Acloque H., Adams M.S., Fishwick K., Bronner-Fraser M., Nieto M.A. (2009). Epithelial-mesenchymal transitions: The importance of changing cell state in development and disease. J. Clin. Investig..

[19] Puisieux A., Brabletz T., Caramel J. (2014). Oncogenic roles of EMT-inducing transcription factors. Nat. Cell Biol..

[20] Kalluri R., Weinberg R.A. (2009). The basics of epithelial-mesenchymal transition. J. Clin. Investig..

[21] Karlsson M.C., Gonzalez S.F., Welin J., Fuxe J. (2017). Epithelial-mesenchymal transition in cancer metastasis through the lymphatic system. Mol. Oncol..

[22] Navas T., Kinders R.J., Lawrence S.M., Ferry-Galow K.V., Borgel S., Hollingshead M.G., Srivastava A.K., Alcoser S.Y., Makhlouf H.R., Chuaqui R. (2020). Clinical Evolution of Epithelial-Mesenchymal Transition in Human Carcinomas. Cancer Res..

[23] Wang Y., Zhou B.P. (2011). Epithelial-mesenchymal transition in breast cancer progression and metastasis. Chin. J. Cancer.

[24] Keegan C.E., Hammer G.D. (2002). Recent insights into organogenesis of the adrenal cortex. Trends. Endocrinol. Metab..

[25] Xing Y., Lerario A.M., Rainey W., Hammer G.D. (2015). Development of adrenal cortex zonation. Endocrinol. Metab. Clin. N. Am..

[26] Rogalla S., Contag C.H. (2015). Early Cancer Detection at the Epithelial Surface. Cancer J..

[27] Mohseny A.B., Hogendoorn P.C. (2011). Concise review: Mesenchymal tumors: When stem cells go mad. Stem Cells.

[28] Bulzico D., Faria P.A.S., Maia C.B., de Paula M.P., Torres D.C., Ferreira G.M., Pires B.R.B., Hassan R., Abdelhay E., Vaisman M. (2017). Is there a role for epithelial-mesenchymal transition in adrenocortical tumors?. Endocrine.

[29] Rubin B., Regazzo D., Redaelli M., Mucignat C., Citton M., Iacobone M., Scaroni C., Betterle C., Mantero F., Fassina A. (2016). Investigation of N-cadherin/beta-catenin expression in adrenocortical tumors. Tumor Biol..

[30] Zeisberg M., Neilson E.G. (2009). Biomarkers for epithelial-mesenchymal transitions. J. Clin. Investig..

[31] Turner N., Grose R. (2010). Fibroblast growth factor signalling: From development to cancer. Nat. Rev. Cancer.

[32] Litvinov S.V., Velders M.P., Bakker H.A., Fleuren G.J., Warnaar S.O. (1994). Ep-CAM: A human epithelial antigen is a homophilic cell-cell adhesion molecule. J. Cell Biol..

[33] Balzar M., Bakker H.A., Briaire-de-Bruijn I.H., Fleuren G.J., Warnaar S.O., Litvinov S.V. (1998). Cytoplasmic tail regulates the intercellular adhesion function of the epithelial cell adhesion molecule. Mol. Cell Biol..

[34] van der Gun B.T., Melchers L.J., Ruiters M.H., de Leij L.F., McLaughlin P.M., Rots M.G. (2010). EpCAM in carcinogenesis: The good, the bad or the ugly. Carcinogenesis.

[35] Loh C.Y., Chai J.Y., Tang T.F., Wong W.F., Sethi G., Shanmugam M.K., Chong P.P., Looi C.Y. (2019). The E-Cadherin and N-Cadherin Switch in Epithelial-to-Mesenchymal Transition: Signaling, Therapeutic Implications, and Challenges. Cells.

[36] Davidson N.E., Sukumar S. (2005). Of Snail, mice, and women. Cancer Cell.

[37] Nieto M.A. (2002). The snail superfamily of zinc-finger transcription factors. Nat. Rev. Mol. Cell Biol..

[38] Olmeda D., Moreno-Bueno G., Flores J.M., Fabra A., Portillo F., Cano A. (2007). SNAI1 is required for tumor growth and lymph node metastasis of human breast carcinoma MDA-MB-231 cells. Cancer Res..

[39] Emadi Baygi M., Soheili Z.S., Essmann F., Deezagi A., Engers R., Goering W., Schulz W.A. (2010). Slug/SNAI2 regulates cell proliferation and invasiveness of metastatic prostate cancer cell lines. Tumour Biol..

[40] Dai S., Zhou Z., Chen Z., Xu G., Chen Y. (2019). Fibroblast Growth Factor Receptors (FGFRs): Structures and Small Molecule Inhibitors. Cells.

[41] Holzmann K., Grunt T., Heinzle C., Sampl S., Steinhoff H., Reichmann N., Kleiter M., Hauck M., Marian B. (2012). Alternative Splicing of Fibroblast Growth Factor Receptor IgIII Loops in Cancer. J. Nucleic. Acids.

[42] Ishiwata T. (2018). Role of fibroblast growth factor receptor-2 splicing in normal and cancer cells. Front. Biosci. (Landmark).

[43] Sbiera S., Sbiera I., Ruggiero C., Doghman-Bouguerra M., Korpershoek E., de Krijger R.R., Ettaieb H., Haak H., Volante M., Papotti M. (2017). Assessment of VAV2 Expression Refines Prognostic Prediction in Adrenocortical Carcinoma. J. Clin. Endocrinol. Metab..

[44] Gazdar A.F., Oie H.K., Shackleton C.H., Chen T.R., Triche T.J., Myers C.E., Chrousos G.P., Brennan M.F., Stein C.A., Larocca R.V. (1990). Establishment and Characterization of a Human Adrenocortical Carcinoma Cell-Line That Expresses Multiple Pathways of Steroid-Biosynthesis. Cancer Res..

[45] Hantel C., Shapiro I., Poli G., Chiapponi C., Bidlingmaier M., Reincke M., Luconi M., Jung S., Beuschlein F. (2016). Targeting heterogeneity of adrenocortical carcinoma: Evaluation and extension of preclinical tumor models to improve clinical translation. Oncotarget.

[46] Kiseljak-Vassiliades K., Zhang Y., Bagby S.M., Kar A., Pozdeyev N., Xu M., Gowan K., Sharma V., Raeburn C.D., Albuja-Cruz M. (2018). Development of new preclinical models to advance adrenocortical carcinoma research. Endocr.-Relat. Cancer.

[47] Franci C., Takkunen M., Dave N., Alameda F., Gomez S., Rodriguez R., Escriva M., Montserrat-Sentis B., Baro T., Garrido M. (2006). Expression of Snail protein in tumor-stroma interface. Oncogene.

[48] Altieri B., Sbiera S., Della Casa S., Weigand I., Wild V., Steinhauer S., Fadda G., Kocot A., Bekteshi M., Mambretti E.M. (2017). Livin/BIRC7 expression as malignancy marker in adrenocortical tumors. Oncotarget.

[49] D’Amici S., Ceccarelli S., Vescarelli E., Romano F., Frati L., Marchese C., Angeloni A. (2013). TNF alpha Modulates Fibroblast Growth Factor Receptor 2 Gene Expression through the pRB/E2F1 Pathway: Identification of a Non-Canonical E2F Binding Motif. PLoS ONE.

[50] Beuschlein F., Weigel J., Saeger W., Kroiss M., Wild V., Daffara F., Libe R., Ardito A., Al Ghuzlan A., Quinkler M. (2015). Major prognostic role of Ki67 in localized adrenocortical carcinoma after complete resection. J. Clin. Endocrinol. Metab..

[51] Shimizu A., Takashima Y., Kurokawa-Seo M. (2002). FGFR3 isoforms have distinct functions in the regulation of growth and cell morphology. Biochem. Bioph. Res. Commun..

[52] Paur J., Nika L., Maier C., Moscu-Gregor A., Kostka J., Huber D., Mohr T., Heffeter P., Schrottmaier W.C., Kappel S. (2015). Fibroblast Growth Factor Receptor 3 Isoforms: Novel Therapeutic Targets for Hepatocellular Carcinoma?. Hepatology.

[53] Zhao Q., Caballero O.L., Davis I.D., Jonasch E., Tamboli P., Yung W.K.A., Weinstein J.N., Shaw K., Strausberg R.L., Yao J. (2013). Tumor-Specific Isoform Switch of the Fibroblast Growth Factor Receptor 2 Underlies the Mesenchymal and Malignant Phenotypes of Clear Cell Renal Cell Carcinomas. Clin. Cancer Res..

[54] Miyamoto Y., Sakane F., Hashimoto K. (2015). N-cadherin-based adherens junction regulates the maintenance, proliferation, and differentiation of neural progenitor cells during development. Cell Adhes. Migr..

[55] Vinson G.P. (2016). Functional Zonation of the Adult Mammalian Adrenal Cortex. Front. Neurosci. (Switz.).

[56] Arnold J. (1866). Ein Beitrag zu der feineren Structur und dem Chemismus der Nebennieren. Virchows. Arch..

[57] Gottschau M. (1883). Struktur und embryonale Entwicklung der Nebennieren bei Säugetieren. Arch. Anat. Physiol..

[58] Chang S.P., Morrison H.D., Nilsson F., Kenyon C.J., West J.D., Morley S.D. (2013). Cell Proliferation, Movement and Differentiation during Maintenance of the Adult Mouse Adrenal Cortex. PLoS ONE.

[59] Vidal V., Sacco S., Rocha A.S., da Silva F., Panzolini C., Dumontet T., Doan T.M.P., Shan J.D., Rak-Raszewska A., Bird T. (2016). The adrenal capsule is a signaling center controlling cell renewal and zonation through Rspo3. Gene Dev..

[60] Walczak E.M., Kuick R., Finco I., Bohin N., Hrycaj S.M., Wellik D.M., Hammer G.D. (2014). Wnt Signaling Inhibits Adrenal Steroidogenesis by Cell-Autonomous and Non-Cell-Autonomous Mechanisms. Mol. Endocrinol..

[61] King P., Paul A., Laufer E. (2009). Shh signaling regulates adrenocortical development and identifies progenitors of steroidogenic lineages. Proc. Natl. Acad. Sci. USA.

[62] Hammer G.D., Basham K.J. (2021). Stem cell function and plasticity in the normal physiology of the adrenal cortex. Mol. Cell Endocrinol..

[63] Lerario A.M., Finco I., LaPensee C., Hammer G.D. (2017). Molecular Mechanisms of Stem/ Progenitor Cell Maintenance in the Adrenal Cortex. Front. Endocrinol..

[64] Hadjidemetriou I., Mariniello K., Ruiz-Babot G., Pittaway J., Mancini A., Mariannis D., Gomez-Sanchez C.E., Parvanta L., Drake W.M., Chung T.T. (2019). DLK1/PREF1 marks a novel cell population in the human adrenal cortex. J. Steroid. Biochem..

