# Supplementary material for: Epithelial and Mesenchymal Markers in Adrenocortical Tissues: How Mesenchymal Are Adrenocortical Tissues?
Source: Cancers (Basel). 2021 Apr 6;13(7):1736. doi: 10.3390/cancers13071736 (PMC8038668; doi:10.3390/cancers13071736)
Supplement: Supplementary file 1 [file cancers-13-01736-s001.pdf]

Supplementary information

Epithelial and mesenchymal markers in adrenocortical tissues: how mesenchymal are adrenocortical tissues?

Iuliu Sbiera[1], Stefan Kircher[2], Barbara Altieri[1], Martin Fassnacht[1,3,4], Matthias Kroiss[1,4,5], Silviu Sbiera[1]

[1] Department of Internal Medicine I, Division of Endocrinology and Diabetes, University Hospital Würzburg, 97080 Würzburg, Germany; [2] Institute for Pathology, University of Würzburg, 97080 Würzburg, Germany; [3] Clinical Chemistry and Laboratory Medicine, University Hospital Würzburg, 97080 Würzburg, Germany; [4] Comprehensive Cancer Center Mainfranken, University of Würzburg, 97080 Würzburg, Germany; [5] Department of Internal Medicine IV, University Hospital Munich, Ludwig-Maximilians-Universität München, 80336 Munich, Germany

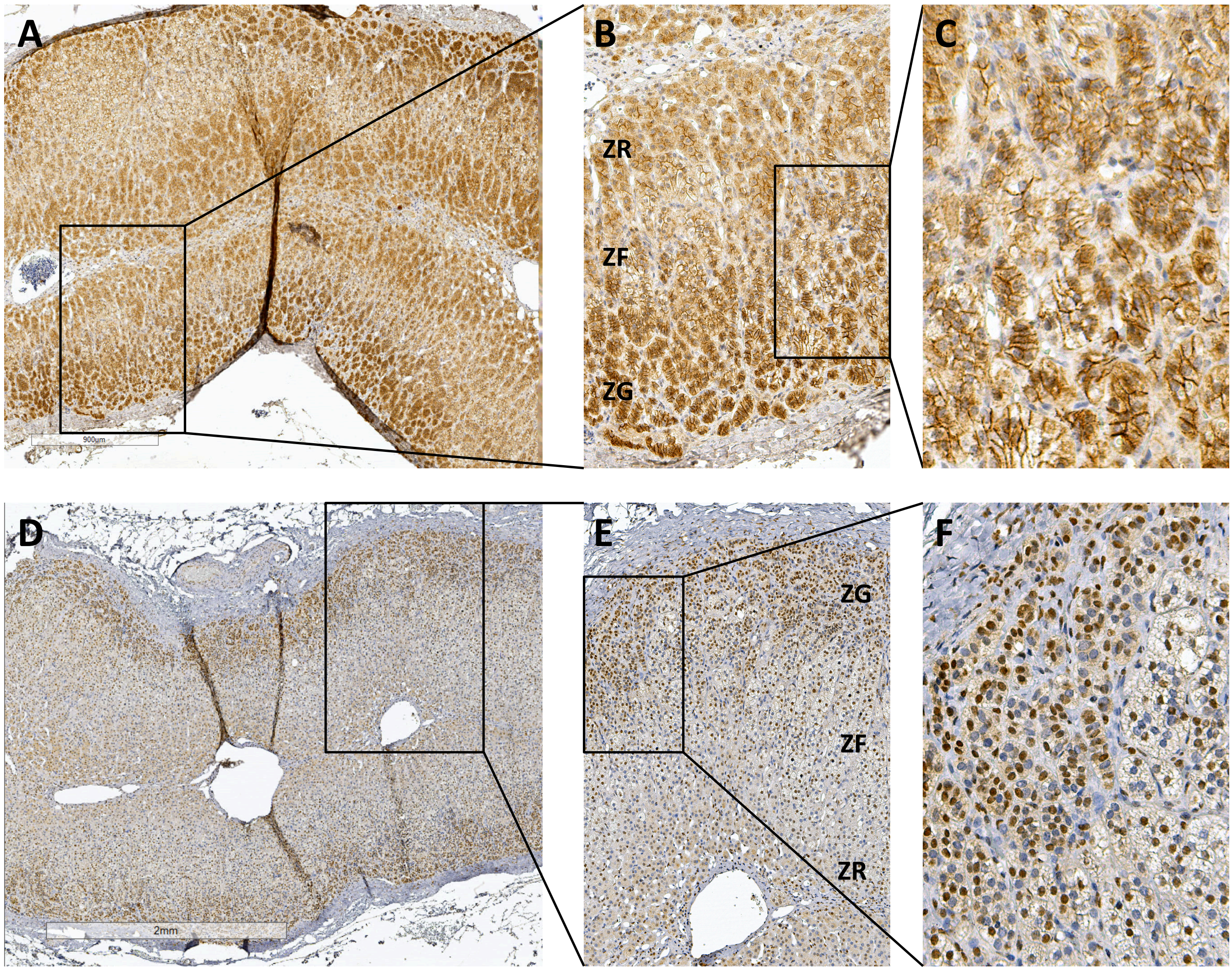

Supplementary Figure 1. Expression of canonical immunohistochemical mesenchymal markers in normal adrenal gland. Staining of mesenchymal markers N-cadherin (A, B and C) and SLUG (D, E and F) in normal adrenal gland. Overview of the middle section of a normal adrenal gland (A and D) with zoom in a representative region (B and E) containing all three functional zones of the adrenal gland (ZG= zona glomerulosa, ZF= zona fasciculata and ZR= zona reticularis) and further zoom to visualize staining subcellular localization in C and F, respectively.
